# Using Qualitative Research to Explore Maternal and Child Health Experiences Among Brazilian Immigrants in the U.S.: A Systematic Review and Meta-Synthesis

**DOI:** 10.3390/ijerph22050759

**Published:** 2025-05-12

**Authors:** Denise Lima Nogueira, Anyelle Barroso Saldanha, Marcia Maria Tavares Machado, Mary L. Greaney, Ana Cristina Lindsay

**Affiliations:** 1 Department of Nursing, Luciano Feijão College, Sobral 62050-215, Brazil; deniseln2009@hotmail.com; 2 Department of Community Health, University Federal of Ceará, Fortaleza 60020-181, Brazil; anyellesaldanha@gmail.com (A.B.S.); marciamachadoufc@gmail.com (M.M.T.M.); 3 Department of Public Health, University Rhode Island, Kingston, RI 02881, USA; mgreaney@uri.edu; 4 Department of Urban Public Health, University of Massachusetts Boston, Boston, MA 02125, USA

**Keywords:** systematic review, qualitative, meta-synthesis, maternal and child health, immigrant, Brazilian, United States

## Abstract

Background: Maternal and child health (MCH) is a critical public health issue affecting individuals, families, and communities worldwide. Immigrant populations, including Brazilian mothers and children in the United States (U.S.), face unique challenges, such as language barriers, limited healthcare access, and socioeconomic disparities that exacerbate health risks. Despite their growing numbers, Brazilian immigrants in the U.S. are an understudied group in MCH research. Objective: This systematic review and qualitative meta-synthesis aims to identify, appraise, and synthesize qualitative and mixed-methods research focused on the MCH experiences of Brazilian immigrants in the U.S. Methods: This review will follow the Preferred Reporting Items for Systematic Review and Meta-Analysis Protocols (PRISMA-P) guidelines and is registered with PROSPERO, an international prospective registry of systematic reviews. Qualitative and mixed-methods research published between 2004 and 2024 that explicitly report qualitative methodology, analysis, and findings related to MCH experiences among Brazilians in the U.S. will be eligible to be included. Studies will be identified through a comprehensive search of seven databases (CINAHL, MEDLINE, PubMed, PsycINFO, Web of Science, Scopus, SocINDEX), and selected according to predefined inclusion/exclusion criteria. Only studies available in English, Portuguese, or Spanish and reporting original qualitative data will be included. Data extraction will be conducted using the Joanna Briggs Institute (JBI) Data Extraction Tool for Qualitative Research. Findings will be synthesized using the JBI meta-aggregation approach in MAXQDA software and evaluated for confidence using the JBI Confidence in Qualitative Research (ConQual) tool. Conclusions: By synthesizing qualitative findings, this review aims to inform the development of culturally responsive healthcare policies, community-based interventions, and future research tailored to the unique needs and experiences of Brazilian immigrant mothers and children in the U.S.

## 1. Introduction

Maternal and child health (MCH) is a cornerstone of public health, profoundly impacting individuals, families, and communities [1,2,3]. The health of mothers and their children is closely intertwined; mothers receiving adequate care before, during, and after pregnancy are more likely to have healthy infants who thrive [1,2,3]. Access to comprehensive MCH services helps reduce mortality and morbidity, while promoting healthy development, educational success, and economic opportunities for both mothers and children [4]. Furthermore, prioritizing MCH initiatives contributes to broader societal well-being, for example, better health during early childhood improves childhood education outcomes, strengthening workforce productivity, and fostering stable family structures. It also reduces long-term healthcare costs and supports sustainable development and social equity [1,2,3,5].

Immigrant mothers are particularly vulnerable during pregnancy and early parenting, facing higher risks of adverse maternal and infant health outcomes compared to non-immigrant populations [6,7,8,9,10]. These risks are often exacerbated by structural barriers such as limited access to healthcare, language differences, immigration status, social exclusion, and financial insecurity [9,10]. Research indicates that immigrant women are more likely to experience delayed or inadequate prenatal care, higher rates of maternal morbidity, and greater challenges in postpartum support [6,7,8,11,12]. Addressing these disparities is essential to improving outcomes and reducing long-term public health burdens.

Research on MCH among immigrant populations is essential, as these communities are often understudied and face unique health challenges such as language barriers, limited access to healthcare, lack of culturally competent care, and socioeconomic disparities [7,8,9,11,12]. Understanding their specific needs will help identify barriers to care and health disparities, informing targeted interventions and policies to improve healthcare access and promote equity [11,12]. Despite the growing body of research on MCH among immigrant populations in the U.S., significant gaps remain—particularly concerning specific subgroups such as Brazilian immigrants. While classified as Latino, Brazilians have unique characteristics that distinguish them in the Latin American context [13,14,15,16]. As the only Portuguese-speaking nation in the region, Brazil’s history has shaped its diverse cultural identity, influenced by indigenous roots and various waves of immigration, including significant contributions from Portugal, Africa, Italy, Germany, etc.

In the U.S., there are over 1,905,000 Brazilians, primarily residing in Massachusetts, Florida, California, New Jersey, New York, etc., enriching their communities’ cultural and economic fabric [15,17,18]. However, like many immigrant populations, they face challenges such as language barriers and limited access to healthcare, making it essential to understand their experiences and needs to offer appropriate interventions [15,17,18,19,20]. Brazilians are frequently excluded from research examining Latino health in the U.S., which often defines “Latino” through a Hispanic or Spanish-speaking lens. As a result, the unique experiences and needs of Brazilian immigrants, particularly around MCH, are often overlooked in national health data, research studies, and public health initiatives. This exclusion contributes to a significant gap in understanding the cultural, linguistic, and systemic factors that shape Brazilian families’ access to and experiences with MCH services. Without focused research, it becomes difficult to design interventions and healthcare services that are culturally and linguistically responsive to this population.

Qualitative methods are essential for exploring health issues in understudied populations, providing rich insights into lived experiences and challenges [21,22,23,24]. Approaches such as interviews and focus groups capture the cultural contexts and social determinants influencing health behaviors [22,25]. This understanding is crucial for developing culturally relevant interventions and policies to address health disparities [22,23,25,26].

Given the size, diversity, and unique characteristics of the Brazilian population in the U.S., there is an urgent need for a comprehensive synthesis of existing qualitative evidence on their MCH experiences. This systematic review and qualitative meta-synthesis aims to address that gap by identifying, appraising, and integrating the available literature on the specific MCH experiences Brazilian immigrants face. This review is guided by the research question: “What are the MCH experiences and challenges faced by Brazilian immigrants in the U.S.?” By synthesizing the findings, this review seeks to generate insights that will inform future research, program development, and policy initiatives tailored to the unique needs of Brazilian immigrant families in the U.S.

## 2. Methods and Analysis

### 2.1. Design

This protocol has been developed and will be reported following the Preferred Reporting Items for Systematic Review and Meta-Analysis Protocols (PRISMA-P) guidelines [27] and the Joanna Briggs Institute (JBI) guidelines for qualitative systematic reviews [28]. The review is registered with the International Prospective Register of Systematic Reviews (PROSPERO) under registration number CRD42024613084 and will be conducted from May to August 2025. This review will focus exclusively on qualitative evidence. Mixed-methods studies will be included regardless of design type (i.e., parallel or sequential designs), as long as they explicitly report identifiable qualitative components that align with the phenomena of interest. Including such studies will capture a broader range of insights. However, only the qualitative findings will be extracted and synthesized. The quantitative component—whether demographic, epidemiological, or survey-based—will be acknowledged but excluded from analysis, ensuring the synthesis remains grounded in qualitative evidence.

### 2.2. Phenomena of Interest

The phenomena of interest is MCH experiences among Brazilians in the U.S. Studies focusing on topics related to maternal health (e.g., pregnancy, prenatal care, childbirth, postpartum care, maternal health behaviors, maternal morbidity and mortality, healthcare access and utilization) and child health (e.g., breastfeeding, child growth and development, nutrition, physical activity, sleep, screen time, child morbidity and mortality, healthcare access and utilization) of Brazilian immigrants living in the U.S. Additional MCH topics that could be examined include maternal mental health (e.g., postpartum depression), childhood vaccination, infectious and non-communicable diseases, trauma, and the influence of immigration-related factors such as legal status, acculturation, or language barriers on MCH outcomes. Only findings generated from qualitative methods will be synthesized, consistent with the review’s qualitative meta-aggregation approach.

### 2.3. Types of Participants

Studies focusing on MCH of Brazilians living in the U.S., conducted with Brazilian parents (mothers, fathers, or legal guardians) of biological or non-biological children, caregivers of Brazilian children, and key informants (e.g., healthcare professionals, faith leaders) who serve, care for, work with or partner with Brazilian families, mothers, and children in the U.S. will be reviewed

### 2.4. Types of Studies

Qualitative and mixed-methods studies that explicitly describe qualitative methods, analysis, and results, conducted with Brazilians residing in the U.S., published in English, Portuguese, or Spanish between 2004 and 2024, and available in full text, will be included. Studies involving key informants (e.g., healthcare professionals such as doctors, nurses, social workers) focusing on any MCH topic related to Brazilians in the U.S. will also be included. For mixed-methods studies, only clearly described qualitative components that meet the inclusion criteria will be extracted and included in the synthesis. These studies may use either parallel or sequential mixed-method designs. There is no restriction on the type of quantitative component, which may include descriptive statistics, health outcomes, or survey results, but these elements will not be analyzed in this review. The focus remains on the qualitative components—such as interviews, focus groups, or ethnographic observations that provide insight into the maternal and child health experiences of Brazilian immigrants. Articles from original mixed-methods studies that do not explicitly describe qualitative methods, analysis, and results, as well as theoretical or methodological articles, will be excluded.

### 2.5. Information Sources

Seven electronic databases will be searched to identify relevant studies: CINAHL, MEDLINE, PubMed, PsycINFO, Web of Science, SocINDEX, and Scopus. These databases were selected to ensure comprehensive coverage of the interdisciplinary literature relevant to MCH among Brazilian immigrants in the U.S. PsycINFO is included to capture studies focusing on psychological and behavioral dimensions of MCH, such as maternal mental health, parenting stress, and acculturation challenges. Scopus was included to broaden the scope of the review by including additional peer-reviewed journals not indexed in other databases. In addition, the reference lists of all included studies will be screened to identify additional eligible sources.

### 2.6. Search Strategy

The search will be conducted from May to August 2025 and limited to studies conducted in the U.S. The search strategy will be based on indexed MESH and APA terms, combined with the Boolean operators “AND” and “OR”, and the use of truncation symbols. The following seven databases will be searched: CINAHL, MEDLINE, PubMed, PsycINFO, Scopus, Web of Science, and SocINDEX. The search will be limited to studies published between 2004 and 2024. This timeframe ensures the comprehensive inclusion of relevant studies reflecting the most current data and trends in MCH among Brazilian immigrants in the U.S. The past two decades have seen significant shifts in both healthcare practices and immigration patterns, making this period particularly relevant for understanding the contemporary MCH experiences and challenges faced by this population. Moreover, it allows the review to focus on studies that are more reflective of the current socio-political and cultural dynamics influencing immigrant health.

The search terms will cover key concepts including the population (e.g., “mother*”, “women”, “fathers”, “child*”, “infant”, “newborn”), health (e.g., “maternal health”, “child health”, “maternal-child health”, “prenatal care”), qualitative research methods (e.g., “qualitative”, “focus group*”, “interview*”, “semi-structured”, “fieldwork”, “key informant”), geographical terms (e.g., “Brazil*”, “Latin*”), location (e.g., “United States”, “U.S.”, “USA”), and immigration (e.g., “Immigrant”). These health-related terms were selected to explicitly reflect the phenomena of interest as defined by the JBI framework—namely, MCH experiences, behaviors, and outcomes among Brazilian immigrants in the U.S.

In addition to database searches, references from included studies will be screened to identify additional studies that the database search may not have captured. The final search results will be consolidated and presented according to the PRISMA diagram for systematic reviews. This approach will ensure a comprehensive and systematic identification of relevant qualitative and mixed-methods studies focused on maternal and child health experiences among Brazilian immigrants in the U.S.

### 2.7. Study Selection

This review will focus on original qualitative or mixed-methods studies conducted in the U.S. on the MCH health of Brazilian immigrants living in the U.S., published in English, Portuguese, or Spanish between 2004 and 2024, and available in full text. Mixed-methods studies that explicitly present qualitative results may also be included.

Two reviewers (DLN and ABS) will independently conduct the study selection process. Covidence software, a tool for supporting and managing systematic reviews, will streamline the search process, organize the review stages, exclude duplicate articles, record the number of studies excluded, and facilitate the review of titles and abstracts to identify eligible studies [29]. Study selection will be conducted independently and in duplicate by the two reviewers to reduce selection bias. If consensus cannot be reached, a third reviewer (MLG) will be consulted to make the final decision.

The reference lists of the eligible studies will also be checked for additional studies. The number and reasons for study exclusions will be recorded in an Excel spreadsheet. A third reviewer will resolve any disagreements during the selection process or any uncertainty about a study’s eligibility after reviewing the title and abstract. The same third reviewer (MLG) will consistently be involved throughout the screening phase to ensure transparency and minimize bias across all decision points.

### 2.8. Risk-of-Bias Assessment

Eligible studies will be assessed for methodological quality using the JBI standard critical appraisal tool for qualitative research. The two researchers (DLN and ABS) will conduct this appraisal independently using a double-blind approach. Studies that meet, at minimum, items 3 and 9 of the JBI tool (Adelaide, Australia) (Available online: https://jbi.global/about-jbi). (accessed on 18 November 2024)—addressing the alignment of methodology and data collection methods and the approval and adherence to ethical research standards, respectively—will be included in the review [28].

After completing the critical appraisal, the researchers will meet to reach a consensus on the results. If consensus is not reached, a third reviewer will decide on the study’s inclusion. The number and reasons for exclusions will be recorded in COVIDENCE.

### 2.9. Data Extraction

Data extraction will be performed using Covidence software, in accordance with JBI’s Qualitative Systematic Review Guide [28]. The JBI Data Extraction and Review Tool (JBI Manual for Evidence Synthesis (2024), Joanna Briggs Institute, Adelaide, Australia) for Qualitative Research will be used independently by two researchers (DLN and ASB). Extracted data will include the study title, journal name, author(s), year of publication, study objectives, study context, geographic location, methodological approach (including study design and data collection methods), sample size, and detailed demographic and socio-cultural information about study participants. This includes the number of participants, type of participants (e.g., mothers, fathers, caregivers, children, healthcare providers, or key informants), and demographic characteristics such as age, gender, immigration status, and socioeconomic background, when available. Information about the study setting or geographic location will also be collected.

To ensure the reliability and accuracy of the data, a detailed data extraction protocol will be followed. This protocol will outline standardized procedures for consistently extracting relevant information across studies. Each set of data extraction forms will be compared for discrepancies, which will be resolved through discussion between the two researchers, with input from a third researcher if necessary. The goal is to reach a consensus on the extracted data, ensuring that all findings are consistently interpreted and accurately reported. This approach enhances the reliability of the extracted data and provides rigor to the synthesis process. Only the qualitative findings from eligible mixed-methods studies will be extracted, ensuring that the synthesis remains focused on the qualitative dimensions of maternal and child health experiences, aligning with the review’s objectives.

### 2.10. Data Synthesis

Findings identified as unequivocal or reliable during data extraction will be synthesized using MAXQDA Analytics Pro 24 software (Berlin, Germany), following the meta-aggregation approach recommended by JBI. MAXQDA is a qualitative data analysis tool used for organizing and analyzing qualitative data [30]. The meta-aggregation approach includes three steps: (1) discovery, (2) categorization, and (3) synthesized discovery [28]. The JBI meta-aggregation approach accommodates studies employing varied qualitative methodologies. This structured synthesis process enables the reconciliation of methodological differences by grouping findings based on their meaning and context rather than the specific analytic techniques used.

In the discovery step, each finding from the included studies will be linked, when possible, to a direct participant quote, fieldwork observation, or other supporting data from the article. In the categorization step, similar findings will be grouped into categories, each accompanied by an explanatory description that captures the full meaning of the group [28]. In the synthesized discovery step, these thematic categories will be merged to form overarching synthesized findings that represent the collective voice across studies. Each synthesized finding will be clearly articulated and supported by illustrative examples from the data, ensuring transparency and traceability. These synthesized findings will then be used to generate actionable recommendations for culturally responsive public policy on maternal and child health for Brazilian immigrant families in the U.S., as well as to identify gaps and priorities for future research.

To address heterogeneity, findings will be organized according to thematic categories reflecting shared and context-specific experiences. Where relevant, themes will be stratified by participant characteristics (e.g., mothers, fathers, healthcare providers), geographic location, or type of health service experienced. This strategy will help illuminate diverse perspectives and ensure a nuanced representation of the Brazilian immigrant experience.

Divergent themes and contradictory findings will be explicitly reported and contextualized to ensure transparency and honor the complexity of participant voices. Results will be presented using a structured narrative format, supported by tables, direct quotes, and visual displays to facilitate accessibility and clarity for researchers, policymakers, and practitioners.

### 2.11. Assessing the Confidence of Findings

To assess the confidence in the findings, the JBI Confidence in Qualitative Research (ConQual) tool will be employed [28]. Each synthesized finding will be appraised based on two criteria: the reliability of the study (classified as high, moderate, low, or very low) and the credibility of the findings, which will be categorized as unequivocal, equivocal, or unsupported [28,31].

Findings classified as unequivocal represent those for which the evidence is clear, consistent, and robust, with little or no ambiguity surrounding the interpretation of the results. Such findings are strongly supported by the data, demonstrating a high degree of certainty in their conclusions. In contrast, equivocal findings are those for which the evidence is less definitive, showing some degree of ambiguity or conflicting results. These findings may require further analysis or additional data to clarify the overall interpretation and draw more robust conclusions.

Only findings classified as unequivocal will be included in the synthesis and presented in the review results. These findings will be systematically reported in tables, figures, and textual summaries, with appropriate consideration of their context and reliability. Findings classified as unsupported, where the evidence does not sufficiently support the conclusions or is of very low quality, will be presented separately and excluded from the final synthesis of results.

## 3. Ethics and Dissemination

As a systematic review, ethical approval for this study is not required. However, only studies that have obtained approval from Human Research Ethics Committees will be eligible to be included. The results of this review will be published in scientific journals and presented at academic conferences.

## 4. Conclusions

While this review protocol outlines a structured approach for synthesizing qualitative evidence on MCH experiences among Brazilian immigrants in the U.S., the actual conclusions will depend on the findings generated through the systematic review process. This synthesis may offer valuable insights to inform culturally responsive healthcare practices, policies, and future research; however, these contributions will remain provisional until the review is completed. Final conclusions will be carefully revisited and aligned with the data and themes that emerge from the synthesis to ensure accuracy, transparency, and relevance.

## Data Availability

No data is available yet as this is a study protocol. Not applicable.

## Abbreviations

The following abbreviations are used in this manuscript:

MCH	Maternal and Child Health
U.S.	United States
JBI	Joanna Briggs Institute
PRISMA-P	Preferred Reporting Items for Systematic Review and Meta-Analysis Protocols
PROSPERO	International Prospective Register of Systematic Reviews
ConQual	Confidence in Qualitative Research
COVIDENCE	Web-based platform that streamlines the process of conducting systematic reviews and other types of literature reviews
